# Feasibility Study of a Time-of-Flight Brain Positron Emission Tomography Employing Individual Channel Readout Electronics

**DOI:** 10.3390/s21165566

**Published:** 2021-08-18

**Authors:** Kuntai Park, Jiwoong Jung, Yong Choi, Hyuntae Leem, Yeonkyeong Kim

**Affiliations:** Molecular Imaging Research & Education (MiRe) Laboratory, Department of Electronic Engineering, Sogang University, Seoul 04107, Korea; hj11113@gmail.com (K.P.); lht0898@gmail.com (H.L.); dusrud026@gmail.com (Y.K.)

**Keywords:** positron emission tomography (PET), brain PET, time-of-flight (TOF), application-specific integrated circuit (ASIC), TOFPET2, coincidence timing resolution (CTR)

## Abstract

The purpose of this study was to investigate the feasibility of a time-of-flight (TOF) brain positron emission tomography (PET) providing high-quality images. It consisted of 30 detector blocks arranged in a ring with a diameter of 257 mm and an axial field of view of 52.2 mm. Each detector block was composed of two detector modules and two application-specific integrated circuit (ASIC) chips. The detector module was composed of an 8 × 8 array of 3 × 3 mm^2^ multi-pixel photon counters and an 8 × 8 array of 3.11 × 3.11 × 15 mm^3^ lutetium yttrium oxyorthosilicate scintillators. The 64-channel individual readout ASIC was used to acquire the position, energy, and time information of a detected gamma ray. A coincidence timing resolution of 187 ps full width at half maximum (FWHM) was achieved using a pair of channels of two detector modules. The energy resolution and spatial resolution were 6.6 ± 0.6% FWHM (without energy nonlinearity correction) and 2.5 mm FWHM, respectively. The results of this study demonstrate that the developed TOF brain PET could provide excellent performance, allowing for a reduction in radiation dose or scanning time for brain imaging due to improved sensitivity and signal-to-noise ratio.

## 1. Introduction

As population aging progresses globally, the importance of early diagnosis and periodic monitoring of brain diseases such as Alzheimer’s disease and Parkinson’s disease is increasing [1,2,3]. Positron emission tomography (PET) is a widely used clinical and research medical imaging device, which is very useful for diagnosing brain diseases as well as various cancers and heart diseases [3,4]. However, conventional whole-body PET is not optimized for brain imaging, resulting in increased radiation dose or scanning time and compromised image quality [3,5,6,7,8]. Therefore, in order to accurately and efficiently diagnose brain diseases, it is necessary to develop a dedicated brain PET with excellent system performance that provides high-quality images.

In order to increase the sensitivity, which depends on the geometric efficiency, the ring diameter of the dedicated brain PET needs to be reduced as much as possible. However, the reduced ring diameter of the dedicated brain PET induces the increased scatter and random coincidence counting rates (CCRs), and the decreased noise equivalent counting rate (NECR) [3,6]. The increases in the scatter and random CCRs add a relatively uniform background to the image, reducing contrast-to-noise ratio, signal-to-noise ratio (SNR), and quantitative accuracy [9,10,11]. In particular, since the random CCR is proportional to the square of the single counting rate, it increases more than the true CCR and scatter CCR. Furthermore, unlike the scatter CCR, it is proportional to the width of the coincidence timing window related to the coincidence timing resolution (CTR) [9]. Therefore, a PET detector with excellent CTR is required to decrease the random CCR.

Time-of-flight (TOF) PET is theoretically possible to determine the location where an annihilation event occurred along the line of response (LOR) using the difference in arrival times of two 511-keV gamma rays detected by a pair of detectors. The location of the occurred annihilation event with respect to the midpoint between the two detectors is given by Δx=c×Δt/2, where *c* is the velocity of light (3 × 10^10^ cm/s) and *Δt* is the time difference. In terms of the clinical performance of a PET, this decreases radiation dose and scanning time and enables more accurate quantification. In terms of the image quality, this improves SNR by reducing the background added to the image. The SNR is roughly proportional to the square root of the NECR assuming that an object is a cylinder with a uniform activity distribution located in the center of the field of view (FOV) and that attenuation, scatter, and random corrections are perfectly applied. If TOF information is incorporated into the analytical image reconstruction algorithm, the estimated TOF SNR gain is equal to $\sqrt{D/\Delta x}$, where *D* is the size of the object. That is, as the CTR proportional to the error of *Δt* improves, the SNR of the image improves due to an increase in the NECR and a decrease in spatial uncertainty (error of *Δx*) [9,11,12,13,14].

The TOF technology with improved CTR should be applied to the dedicated brain PET with the reduced ring diameter to improve SNR and quantitative accuracy and to achieve meaningful TOF gain (SNR and NECR). Previously, we determined an optimal geometry by evaluating the performance of a TOF brain PET such as sensitivity, NECR, and spatial resolution while changing the ring diameter and scintillator thickness using Monte Carlo simulation. The determined ring diameter and scintillator thickness were 257 and 15 mm, respectively [15]. In addition, a TOF PET detector with optimized optical properties was developed by evaluating the performance of the detector (photopeak position, energy resolution, and CTR) while changing the surface treatment and reflective materials of the scintillator. The scintillator of the developed detector was mechanically polished except for a roughening entrance surface. The entrance surface of the scintillator was optically isolated by Teflon tape. In addition, all lateral surfaces were optically isolated by both enhanced specular reflector (ESR) film and Teflon tape [16].

The CTR is mainly determined by the physical properties of the PET detector related to light generation, light transport, and light conversion. However, due to readout electronics, the CTR deteriorates as the output signal of a silicon photomultiplier (SiPM) corresponding to the detected gamma ray is distorted or the amplitude of noise increases. The improved CTR can be achieved using individual channel readout electronics that minimize distortion of the output signal in comparison to a multiplexing circuit based on the method such as charge division or signal delay [11,17,18,19,20,21,22,23,24]. Therefore, the fast TOF PET could be achieved using low-noise individual channel readout electronics that allow for fast TOF measurements as well as a PET detector with excellent CTR.

The purpose of this study was to investigate the feasibility of a TOF brain PET providing high-quality images. This paper describes the overall system configuration of the developed TOF brain PET including the individual channel readout electronics as well as the geometry and detector. It also presents the performance evaluation of the conceptual brain PET to verify the feasibility of the proposed TOF PET using a point source and a phantom.

## 2. Materials and Methods

### 2.1. Geometry of the TOF Brain PET

**Figure 1:**
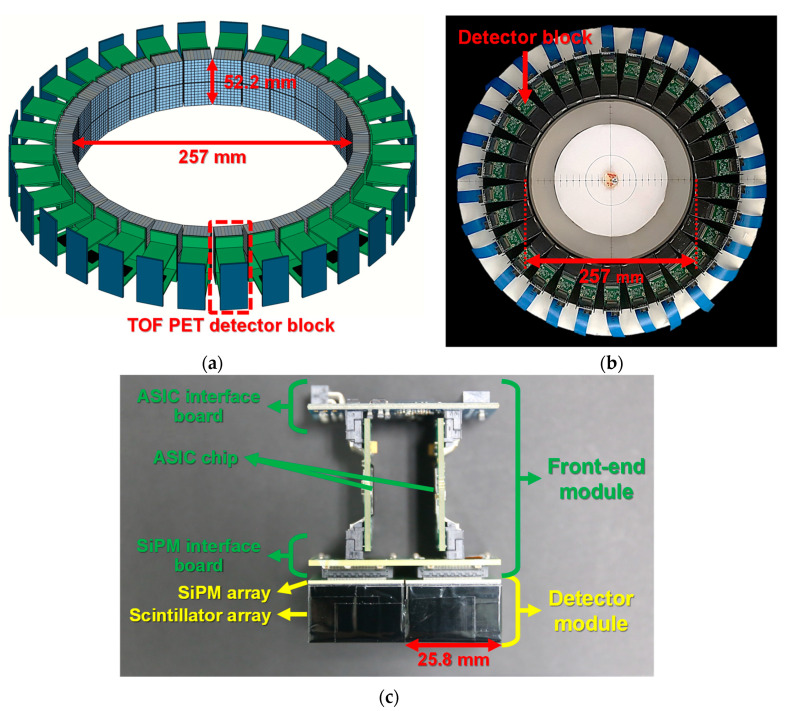
(**a**) Geometry of the proposed TOF brain PET. (**b**) Photograph of the developed TOF brain PET consisting of 30 detector blocks connected to flexible flat cables. (**c**) Configuration of the TOF PET detector block.

The developed TOF brain PET consisted of 30 detector blocks with 3840 channels placed in a circle on a custom-made acetal gantry. The 30 detector blocks were arranged at equal angular intervals of 12 degrees in a ring with an axial FOV of 52.2 mm, as shown in Figure 1a,b. The ring diameter was chosen to be 257 mm to improve sensitivity [15]. A transaxial FOV of the developed TOF brain PET was approximately 181 mm, covering up to the maximum size of the human brain [25].

Figure 1c shows the configuration of the TOF PET detector block. Each detector block was composed of two detector modules and a front-end module (FEM) consisting of two boards equipped with an application-specific integrated circuit (ASIC) chip, a SiPM interface board, and an ASIC interface board. The two detector modules, each of which had an area of 25.8 × 25.8 mm^2^, were axially connected to the SiPM interface board with an axial gap of 0.6 mm. The two ASIC boards were assembled almost perpendicularly to both the SiPM interface board and the ASIC interface board.

### 2.2. TOF PET Detector

**Figure 2:**
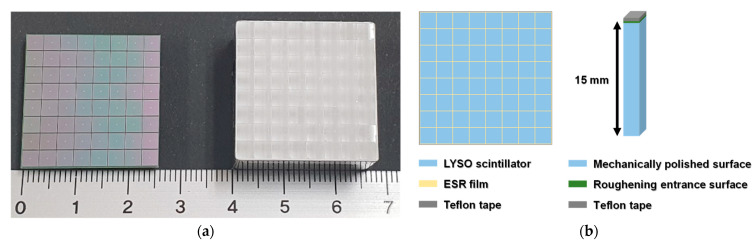
(**a**) 8 × 8 array of 3 × 3 mm^2^ MPPCs (left) and 8 × 8 array of 3.11 × 3.11 × 15 mm^3^ LYSO scintillators (right). (**b**) Design of the LYSO array considering optimal optical properties (surface treatment and reflective materials).

The TOF PET detector module was composed of an 8 × 8 array of 3 × 3 mm^2^ multi-pixel photon counters (MPPCs) (S13361-3050AE-08, Hamamatsu Photonics K.K., Hamamatsu, Japan) and an 8 × 8 array of 3.11 × 3.11 × 15 mm^3^ lutetium yttrium oxyorthosilicate (LYSO) scintillators (Crystal Photonics, Inc., Sanford, FL, USA), as shown in Figure 2a. The thickness of the LYSO was chosen as 15 mm by considering the trade-off among sensitivity, TOF capabilities, and parallax error [15].

Figure 2b shows the design of the LYSO array. To achieve excellent CTR, each LYSO was mechanically polished except for a roughening entrance surface. All lateral surfaces and the entrance surface of the LYSO were optically isolated by ESR film and Teflon tape, respectively. In addition, all lateral surfaces of the LYSO array were optically isolated by Teflon tape [16].

The MPPC and LYSO arrays were coupled using a silicone adhesive (3145 RTV, The Dow Chemical Company, Midland, MI, USA) with a refractive index of 1.49 at 430 nm to match the refractive index between optical boundaries (MPPC: 1.55 at 450 nm, LYSO: 1.82 at 420 nm) taking Snell’s law into account. In addition, this optical coupling material was used to improve the collection efficiency of scintillation light and to maintain its adhesion and optical properties for a long time [26,27].

### 2.3. Individual Channel Readout Electronics

**Figure 3:**
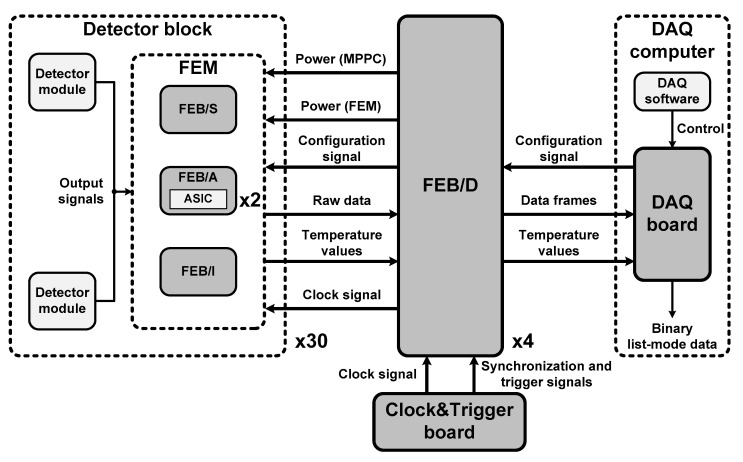
Block diagram of the individual channel readout electronics for the developed TOF brain PET.

The individual channel readout electronics were based on a 64-channel commercial ASIC (TOFPET2, PETsys Electronics S.A., Oeiras, Portugal) to enable fast TOF measurement [28]. Figure 3 shows a block diagram of the individual channel readout electronics for the developed TOF brain PET. They were composed of the FEM, a front-end board (FEB) for data acquisition (DAQ) called FEB/D, a DAQ board, and a Clock&Trigger board.

#### 2.3.1. Front-End Module (FEM)

As described in Section 2.1, the main components of the FEM were the ASIC board, called FEB/A, the SiPM interface board, called FEB/S, and the ASIC interface board called FEB/I [29]. FEB/A was equipped with the TOFPET2 ASIC chip, a temperature sensor, and two connectors to plug in FEB/S and FEB/I. The ASIC were low-power and low-noise electronics providing readout and digitization of signals from 64 individual channels to acquire the position, energy, and time information of an interacted gamma ray in each scintillator.

**Figure 4:**
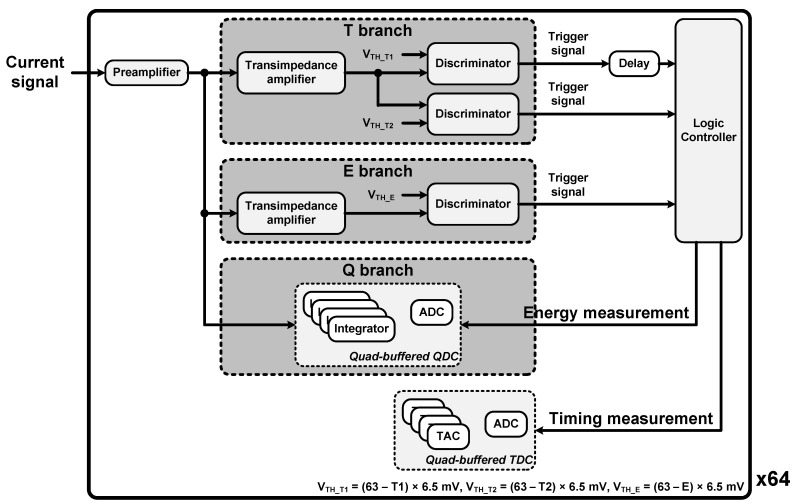
Block diagram of TOFPET2 ASIC in QDC mode.

As shown in Figure 4, it consisted of a preamplifier, two transimpedance amplifiers, three fast discriminators, a quad-buffered charge-to-digital converter (QDC), and a quad-buffered dual ramp time-to-digital converter (TDC) for each channel [30]. The output current signal of the detector module passed through the preamplifier, a current conveyor with low input impedance, and was then replicated to three branches: T, E, and Q. The current signals of the T and E branches were converted into voltage signals by transimpedance amplifiers, and that of the Q branch was digitized by the QDC comprising four integrators and a 10-bit Wilkinson analog-to-digital converter (ADC) for energy measurement.

The voltage signal of the T branch was transferred to two discriminators each having a threshold voltage value, called V_TH_T1_, for time measurement and a threshold voltage value, called V_TH_T2_, for rejecting the dark count and starting the charge integration window. The TDC, which comprised four time-to-amplitude converters (TACs) and a 10-bit Wilkinson ADC, was used for time measurement. The voltage signal of the E branch was transferred to a discriminator with a threshold voltage value, called V_TH_E_, for rejecting low voltage signal. All threshold voltage values were set by DAQ software as digital values, called T1, T2, and E, as follows:

V_TH_T1_ = (63 − T1) × 6.5 mV (1)

V_TH_T2_ = (63 − T2) × 6.5 mV (2)

V_TH_E_ = (63 − E) × 6.5 mV (3)

Trigger signals output from these discriminators were transferred to a logic controller. The logic controller activated the QDC and TDC under certain conditions and determined that the detected gamma ray was a valid event when all trigger signals were on the rising edge. The raw data, including information about valid events, were transmitted to the ASIC interface board through four LVDS output links with a maximum data rate per link of 800 Mbit/s and a maximum output event rate per channel was 0.6 kevent/s. The specifications of the ASIC are summarized in Table 1.

FEB/S was equipped with connectors for transmission of 128 output signals of two MPPC arrays to two FEB/As, and two sensors for monitoring of the temperature of two MPPC arrays. FEB/I managed the communication between the two FEB/As and FEB/D through a 50 cm-long flexible flat cable connected to an on-board FEM port.

#### 2.3.2. Front-End Board for Data Acquisition (FEB/D)

**Figure 5:**
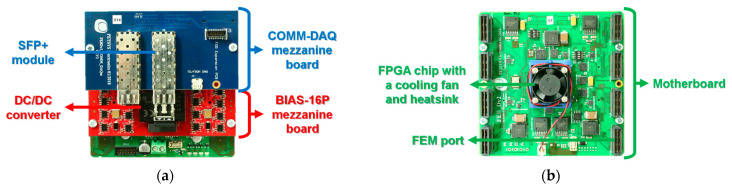
Photographs of FEB/D capable of connecting up to eight FEMs: front (**a**) and back (**b**) views.

Figure 5 shows FEB/D comprising a motherboard, a BIAS-16P mezzanine board, and a COMM-DAQ mezzanine board [29,31]. The motherboard was equipped with a field programmable gate array (FPGA) chip, and eight FEM ports for operating up to eight FEMs through 50 cm-long flexible flat cables. The role of the motherboard was to distribute power, clock, and configuration signals to each FEM, to receive temperature values and raw data on the ASIC from each FEM, and to collect raw data into data frames, as shown in Figure 3. The motherboard only required a single external supply voltage of 12 V with a maximum current of 4 A and used a set of switching regulators to supply power to eight FEMs as well as the entire FEB/D.

The BIAS-16P mezzanine board supplied voltage to 16 MPPC arrays through two on-board DC/DC converters, each with a maximum current of 20 mA. Each DC/DC converter provided eight positive voltage lines capable of independent voltage settings within the range 0–72 V by DAQ software, with an average current per line of 2.5 mA.

The COMM-DAQ mezzanine board was equipped with an enhanced small form-factor pluggable (SFP+) optical transceiver module for transmission of data frames to the DAQ board and receipt of configuration signals from the DAQ board. The communication between the COMM-DAQ mezzanine board and the DAQ board was through the SFP+ high-speed serial optical link. The maximum output event rate per link was 100 Mevent/s at up to 6.6 Gbit/s. In addition, the COMM-DAQ mezzanine board was equipped with a small multiple connector type Q (SMC-Q) for receipt of clock, synchronization, and trigger signals from the Clock&Trigger board through a 2 m-long flat cable assembly.

#### 2.3.3. DAQ Board and Clock&Trigger Board

**Figure 6:**
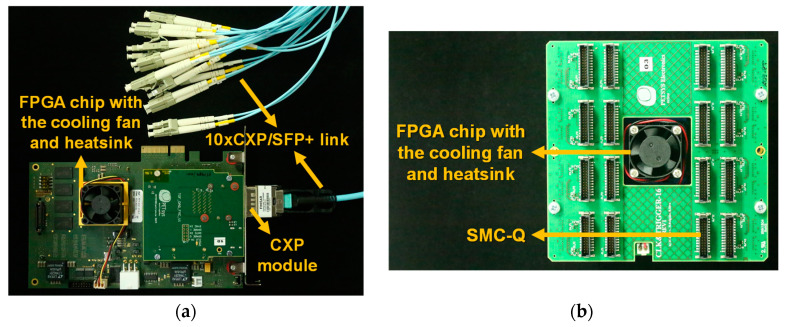
Photographs of the DAQ board (**a**) and the Clock&Trigger board (**b**). For the developed TOF brain PET, a 10xCXP/SFP+ link was used to communicate with the DAQ board.

As shown in Figure 6a, the DAQ board was equipped with an FPGA chip and a CoaXPress (CXP) optical transceiver module capable of connecting up to 12 SFP+ modules through a 5 m-long 12xCXP/SFP+ link [32]. The DAQ board was mounted on a PCIe 2.0 x4 slot on a computer to be operated and controlled. The role of the DAQ board was to generate signals for ASIC configuration, to run the calibration process for transimpedance amplifiers, discriminators, QDCs, and TDCs, to receive data frames from FEB/Ds, to store them on the computer, and to convert them into binary list-mode data, as shown in Figure 3. The DAQ board also monitored the values of the temperature sensors mounted on FEB/S and FEB/A. All of these functions were controlled by DAQ software run on 64-bit CentOS 7 Linux [33]. The maximum output event rate from the DAQ board to the computer was 250 Mevent/s.

Figure 6b shows the Clock&Trigger board comprising the same motherboard and COMM-DAQ mezzanine board as FEB/D and 16 SMC-Qs [32]. The role of the Clock&Trigger board was to generate the system reference clock of 200 MHz, synchronization, and trigger signals and to send them to up to 16 FEB/Ds, as shown in Figure 3. All boards equipped with FPGA chips were programmed with bit files implemented to perform the functions of each board through the standard JTAG interface. All of the FPGA chips were Kintex-7 FPGAs (XC7K160T, Xilinx, Inc., San Jose, CA, USA). In addition, each of these boards was equipped with a cooling fan and heatsink for dissipation of the heat from the FPGA chip.

### 2.4. Performance Evaluation

Before evaluating the feasibility of the TOF brain PET employing individual channel readout electronics, the ASIC calibration process was performed at room temperature with a breakdown voltage of the MPPC set to 53 V. All experiments were also carried out at room temperature and the TOF PET detector blocks were cooled using air circulation. The binary list-mode data acquired using the developed TOF brain PET were sorted into prompt coincidence data considering the energy window of 10%, the coincidence timing window of 5 ns (without time offset correction), and all possible LORs across the FOV. The acquired energy spectra were not corrected for the energy nonlinearity resulting from the SiPM saturation and the nonlinear QDC response. All tomographic images were nonTOF images reconstructed without attenuation, normalization, scatter, and random corrections.

#### 2.4.1. Energy Resolution, Coincidence Counting Rate, and Coincidence Timing Resolution

**Figure 7:**
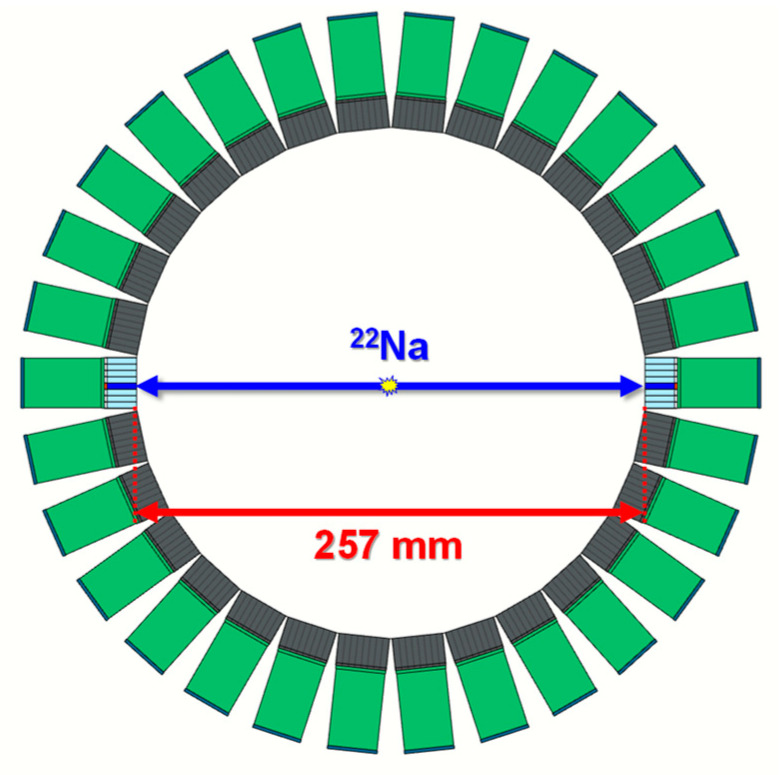
Experimental setup to evaluate the performance (energy resolution, CCR, and CTR) of the detector module. The ^22^Na point source was placed at the center between a pair of channels.

As shown in Figure 7, a 1.8 MBq of ^22^Na point source having an active diameter of 1 mm was placed at the center between a pair of channels, and the data were acquired using the pair of channels of two detector modules facing each other on the TOF brain PET for 240 s.

The energy resolution was measured by calculating the full width at half maximum (FWHM) using a Gaussian fit to the photopeak position corresponding to 511 keV in the energy spectrum acquired with energy values of the list-mode data. The CCR was measured by calculating the total number of prompt coincidence data detected during the data acquisition time. The CTR was measured by calculating the FWHM using a Gaussian fit to the coincidence peak position in the time spectrum acquired with the difference in time values of the prompt coincidence data.

To optimize the performance of the detector module, the energy resolution, CCR, and CTR were measured (repeated three times) while changing the overvoltage (V_OVER_) of the MPPC and T1 of the ASIC. The V_OVER_ and T1 values were set at 1 V increments from 1 to 7 V and five increments from 20 to 60, respectively. The optimal V_OVER_ and T1 values were determined considering average values of the energy resolution, CCR, and CTR.

#### 2.4.2. Spatial Resolution

In order to evaluate the spatial resolution of the TOF brain PET, the data were acquired using the 1.7 MBq of ^22^Na point source placed at the center of the FOV for 240 s. In addition, the data acquisition was repeated while moving the same point source to the following transaxial distances from the center: 20, 40, 60, and 80 mm. The tomographic images of the point source were reconstructed by a 2-dimensional filtered backprojection algorithm with a Hann filter (cut-off frequency = 0.42 × Nyquist frequency) and 514 × 514 pixels of 0.5 × 0.5 mm^2^. The spatial resolution was measured by calculating the FWHM using a Gaussian fit to each pixel peak position in response functions (1-dimensional profiles) acquired with the reconstructed images of the point source.

#### 2.4.3. Phantom Imaging

The data were acquired using a custom-made hot-rod phantom filled with 10.4 MBq of ^18^F placed at the center of the FOV to evaluate the imaging capability of the TOF brain PET for 120 s. The diameter and height of the hot-rod phantom were 75 and 40 mm, respectively, and it consisted of 40 rods with different diameters (2.5, 3.5, 4.5, 5.5, and 6.5 mm). The tomographic images of the hot-rod phantom were reconstructed by a 3-dimensional maximum-likelihood expectation-maximization algorithm with 31 iterations and 514 × 514 × 514 voxels of 0.5 × 0.5 × 0.5 mm^3^.

## 3. Results

### 3.1. Energy Resolution, Coincidence Counting Rate, and Coincidence Timing Resolution

**Figure 8:**
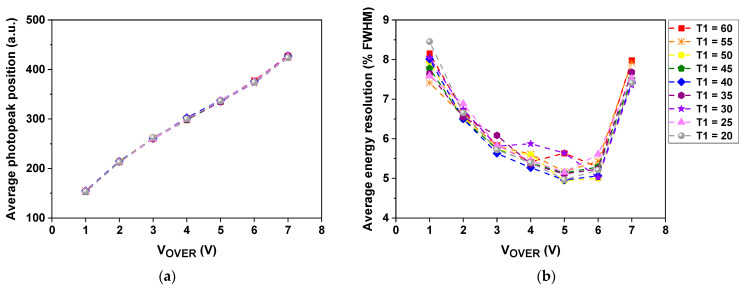

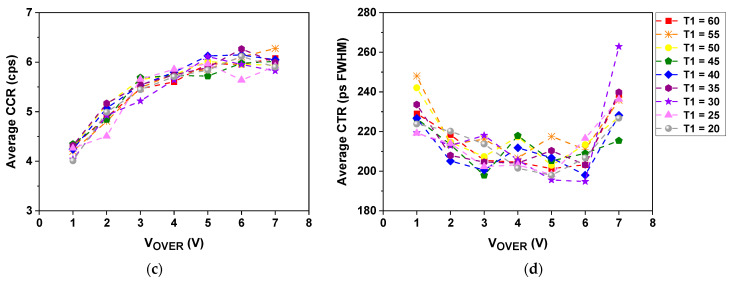
Average photopeak position (**a**), energy resolution (**b**), CCR (**c**), and CTR (**d**) measured using a pair of channels at different V_OVER_ and T1 values.

Figure 8 shows average values of the photopeak position, energy resolution, CCR, and CTR measured using a pair of channels at different V_OVER_ and T1 values. The average photopeak position was almost linearly shifted to higher QDC values with increasing V_OVER_ value, regardless of the T1 value (Figure 8a). The average energy resolution gradually improved as the V_OVER_ value increased, but rapidly deteriorated after the V_OVER_ value of 5–6 V (Figure 8b). The average CCR increased, but the rate of change gradually decreased as the V_OVER_ value increased (Figure 8c). In addition, like the average energy resolution, the effect of the T1 value on the average CCR was relatively small. The average CTR improved as the V_OVER_ value increased, but rapidly deteriorated after the V_OVER_ value of 5–6 V (Figure 8d). At the V_OVER_ values of 5 and 6 V (T1 = 30), the average CTRs were 195.5 ± 9.2 and 194.7 ± 12.1 ps FWHM, respectively.

**Figure 9:**
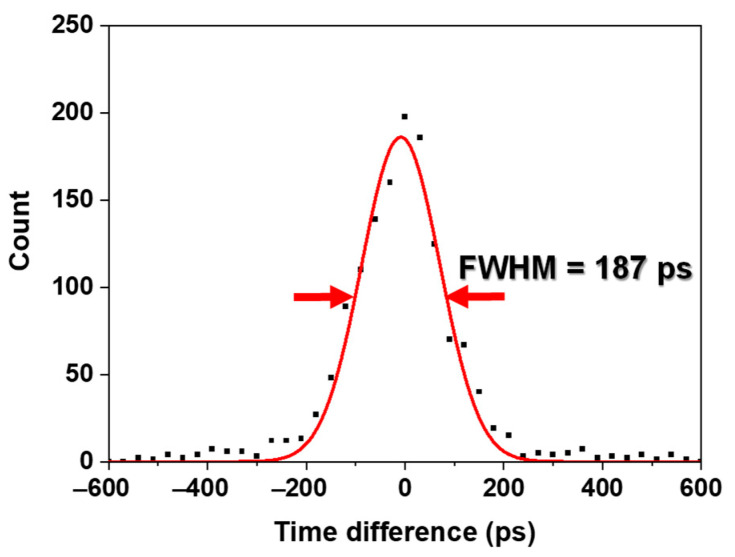
Time spectrum acquired at the V_OVER_ of 5 V and T1 of 30. The solid line was the result of applying a Gaussian fit to the coincidence peak position.

The optimal V_OVER_ and T1 values were determined to be 5 V and 30, respectively, considering the standard deviation of the CTR. The average values of the photopeak position, energy resolution, and CCR were 335 ± 3, 5.6 ± 1.4% FWHM (without energy nonlinearity correction), and 6.1 ± 0.2 cps, respectively at the optimal values. The best CTR of 187 ps FWHM was achieved, as shown in Figure 9.

**Figure 10:**
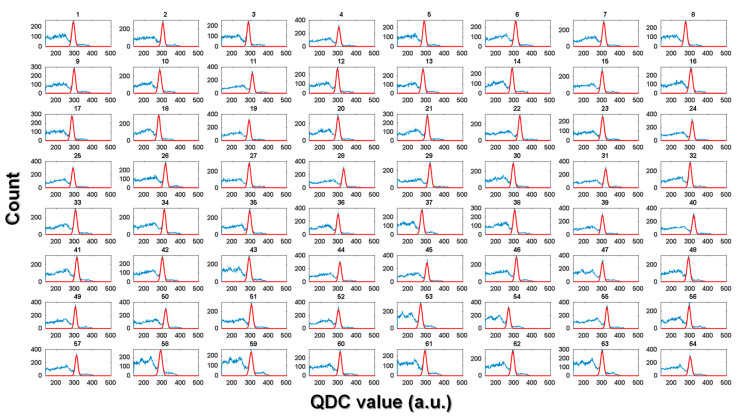
Representative energy spectra acquired from 64 channels of the TOF brain PET. The thick solid lines were the result of applying a Gaussian fit to the photopeak position corresponding to 511 keV.

Figure 10 shows the representative energy spectra of 64 channels (one of 60 detector modules) among the energy spectra of 3840 channels acquired with the TOF brain PET using the ^22^Na point source placed at the center of the FOV to evaluate the spatial resolution. The average energy resolution and CCR of the 3840 channels were 6.6 ± 0.6% FWHM (not corrected for the energy nonlinearity) and 13.3 kcps at 1.7 MBq, respectively.

### 3.2. Spatial Resolution

**Figure 11:**
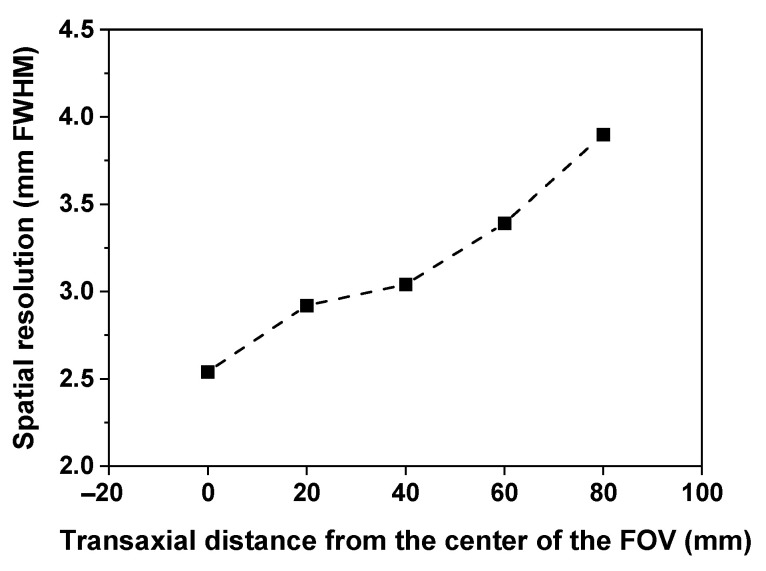
Spatial resolution of the TOF brain PET as a function of the transaxial distance from the center of the FOV.

Figure 11 shows the spatial resolution of the TOF brain PET as a function of the transaxial distance from the center of the FOV. A transaxial spatial resolution of 2.5 mm FWHM was achieved at the center of the axial and transaxial FOV.

### 3.3. Phantom Imaging

**Figure 12:**
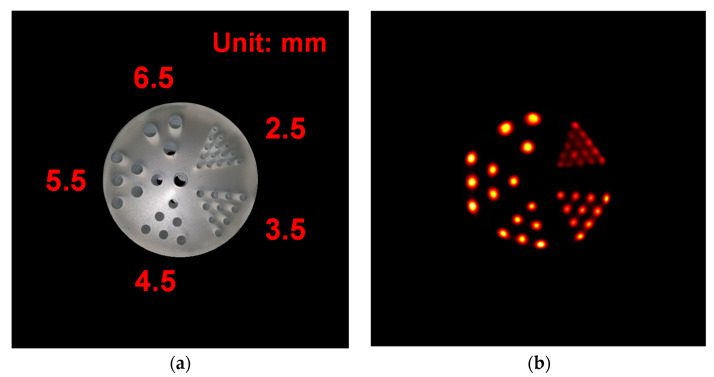
(**a**) Photograph of the hot-rod phantom comprising 40 rods with different diameters (2.5, 3.5, 4.5, 5.5, and 6.5 mm). (**b**) Transverse slice of the hot-rod phantom image acquired using the TOF brain PET.

Figure 12 shows a tomographic image of the hot-rod phantom with a bit depth of 8-bit acquired using the TOF brain PET. The rods were clearly resolved down to a diameter of 2.5 mm in the hot-rod phantom image.

## 4. Discussion

In this study, we have developed a TOF brain PET by employing an optimal geometry to improve sensitivity, a fast TOF PET detector to achieve excellent CTR, and individual channel readout electronics to enable TOF measurement. The feasibility of the TOF brain PET has been evaluated by measuring the energy resolution, CCR, CTR, and spatial resolution. Tomographic images of the custom-made hot-rod phantom have been acquired to evaluate the imaging capability of the TOF brain PET. The image quality phantoms specified in the national electrical manufacturers association (NEMA) standards were not used because it is not fully functional but a proof-of-principle system demonstrating the feasibility of the TOF brain PET. The developed TOF brain PET has the potential to improve sensitivity, spatial resolution, and image quality compared to a conventional whole-body PET in brain imaging due to the reduced ring diameter, the detector size, and the TOF capabilities. It is expected that the TOF brain PET will provide better system performance than a conventional PET employing a multiplexing circuit that reduces the number of readout channels but degrades the overall performance of the detector.

The arrival time of the detected gamma ray was measured by a leading-edge discrimination method using the discriminator in the individual channel readout electronics, which could cause timing walk, an energy-dependent timing variation, leading to degradation of the CTR [9,34]. There are two general ways to reduce it. One is to increase the amplitude of the output signal of the SiPM input to the discriminator, and the other is to decrease the threshold voltage value of the discriminator. In general, for the former, it is necessary to increase the operating voltage of the SiPM or the gain of the amplifier, which causes an increase in the dark count or the amplitude of noise. In the latter case, as the value continues to decrease, the arrival time of the dark count rather than the detected gamma ray may be measured. Therefore, in order to reduce the extent of CTR degradation due to timing walk and dark count, the optimal V_OVER_ and T1 were determined from the results shown in Figure 8. The data showed that the best CTR of 187 ps FWHM was achieved at the V_OVER_ of 5 V and T1 of 30. Although the materials and methods used for the measurement were not the same, this result was superior to that measured with commercial or prototype PET systems reported to date (210–544 ps), given 15 mm LYSO:Ce (not co-doped with Ca), analog SiPM, and measurement at room temperature [11,17,35,36]. This is due to the optimization of the TOF PET detector module and the use of individual channel readout electronics with a TDC resolution of 20 ps.

It was possible to clearly distinguish all rods in the tomographic image of the hot-rod phantom and the diameter of the smallest among these was the same as the transaxial spatial resolution measured at the center of the FOV. As shown in Figure 11, the spatial resolution of the developed brain PET was degraded toward the outside of the FOV due to an increase in parallax error caused by the reduced ring diameter [3,6]. If TOF technology is applied, it is predicted that the degradation of the spatial resolution can be improved due to allowing the use of smaller voxels in the image reconstruction [14]. In addition, it is expected that the image quality will be greatly improved due to excellent CTR.

Since the scintillator thickness of 15 mm is shorter than that of most commercial PET systems, although the sensitivity is reduced due to a decrease in detection efficiency, the spatial resolution is improved due to a decrease in parallax error. Additionally, important benefits can be obtained due to TOF capabilities achieved by using the reduced thickness [9,10,15,17]. If the tomographic image of an object having the same diameter as the transaxial FOV is acquired using the TOF brain PET with the CTR of 187 ps, the TOF SNR gain would be about 2.5 under the following assumptions: the object is a cylinder with a uniform activity distribution located in the center of the FOV and attenuation, scatter, and random corrections are perfectly applied. Considering the relationship between SNR and NECR, the radiation dose or scanning time could be reduced by about 6.5-fold which allows the radiation dose to reduce to about 1 mSv for a typical ^18^F-fluorodeoxyglucose (FDG)-PET study.

The performance of the MPPC array and ASIC chip in the TOF PET detector block deteriorates as the operating temperature increases due to the heat generated by both of those components as well as the heat transferred from the ASIC chip to the MPPC array [18,30]. Moreover, if the temperature of the detector block is different during the ASIC calibration process and the data acquisition process, the performance would be nonuniform [33]. As shown in Figure 8d, the average CTRs acquired at different V_OVER_ and T1 values did not have a monotonic relationship due to this temperature difference. An effective cooling method is required to constantly maintain the temperatures of all of the MPPCs and ASIC chips low and similar, which enables the TOF PET detector blocks to provide improved and uniform performance.

We are currently constructing a prototype TOF brain PET with an extended axial FOV applying a water-cooled system. A further study will be performed to correct the time offset required for TOF image reconstruction, to evaluate the performance of the TOF brain PET according to the NEMA standards, and to acquire high-performance TOF PET images.

## 5. Conclusions

We have developed and evaluated the performance of a proof-of-principle TOF brain PET with the geometry optimized for brain imaging by employing a PET detector with optimized optical properties and individual channel readout electronics. The results of this study demonstrate that the developed TOF brain PET could provide excellent performance, allowing for the reduction in radiation dose or scanning time for brain imaging due to improved sensitivity and SNR.

## Figures and Tables

**Table 1:** Brief specifications of the ASIC.

Description	TOFPET2 (Version 2c)
Technology	110 nm CMOS
Supply voltage	1.2 V, 2.5 V
Power consumption	8.2 mW/channel
Main clock frequency	400 MHz
TDC clock frequency	200 MHz
TDC time bin	31 ps
TDC resolution	20 ps (r.m.s.)
QDC dynamic range	0–1500 pC
Maximum data rate	3.2 Gbit/s
Maximum output event rate	40 Mevent/s

## Data Availability

The data presented in this study are not publicly available due to ongoing results protection with respect to a prototype time-of-flight brain positron emission tomography with an extended axial field of view applying a water-cooled system we are currently constructing.
